# Granulomatöse Polyangiitis mit neurologischer Erstmanifestation

**DOI:** 10.1007/s00115-020-00966-1

**Published:** 2020-07-28

**Authors:** Erik Ellwardt, Frank Birklein

**Affiliations:** Klinik und Poliklinik für Neurologie, Universitätsmedizin der Johannes Gutenberg Universität Mainz, Langenbeckstraße 1, 55131 Mainz, Deutschland

## Hintergrund

Die Granulomatose mit Polyangiitis (GPA; früher auch als Wegener-Granulomatose bezeichnet) ist eine seltene rheumatologische Erkrankung der Gefäße mit einhergehender Granulombildung der oberen und unteren Atemwege. Männer und Frauen sind etwa gleich häufig betroffen und der Erkrankungsgipfel liegt um das 50. Lebensjahr [1]. Durch die Gefäßentzündung können prinzipiell alle Organe betroffen sein. In abnehmender Häufigkeit sind jedoch meistens folgende Organsysteme betroffen: Nieren, obere und untere Atemwege, Ohren, Augen, Haut, Herz und Nervensystem (peripheres [PNS] und zentrales Nervensystem [ZNS]; [2]). Eine Erstmanifestation einer GPA mit einer Schädigung des peripheren oder zentralen Nervensystems ist selten, im Verlauf können jedoch bei bis zu 50 % der GPA-Patienten neurologische Symptome auftreten [3, 4]. Wir berichten hier von einer 60-jährigen Patientin, die sich mit einer seit ca. 4 Wochen bestehenden Gangstörung und Schwankschwindel, einer Hörminderung und Sehstörungen in unserer neurologischen Poliklinik vorstellte.

## Fallbeschreibung

Die 60-jährige Patientin stellte sich aufgrund der Gangstörung und des Schwankschwindels vor. Seit 4 Wochen bestand eine langsam zunehmende Gangstörung einhergehend mit brennenden Dysästhesien an den Fußsohlen sowie einer leichten Fußheberschwäche (Kraftgrad 4/5) beider Seiten. Die Achillessehenreflexe waren beidseits nicht auslösbar. Eine Gangataxie oder Koordinationsstörung bestanden nicht. Die Patientin gab zusätzlich an, seit einer Woche vertikal und horizontal versetzte Doppelbilder sowie eine Hörminderung auf dem rechten Ohr zu haben. Die klinisch neurologische Untersuchung war diesbezüglich bis auf einen Blickrichtungsnystagmus nach rechts initial unauffällig, die subjektiven Beschwerden konnten klinisch nicht eindeutig nachvollzogen werden. In der erweiterten Anamnese berichtete die Patientin von einem seit 3 Monaten anhaltenden chronischen Schnupfen. Laborchemisch fiel bereits ein deutlich erhöhtes CRP auf (133 mg/l, Normwert <5 mg/l). Zu diesem Zeitpunkt hatten wir den Verdacht auf eine vaskulitische Polyneuropathie (PNP) und eine zentrale Beteiligung im Rahmen einer GPA und es erfolgte die stationäre Abklärung.

**Figure 1:**
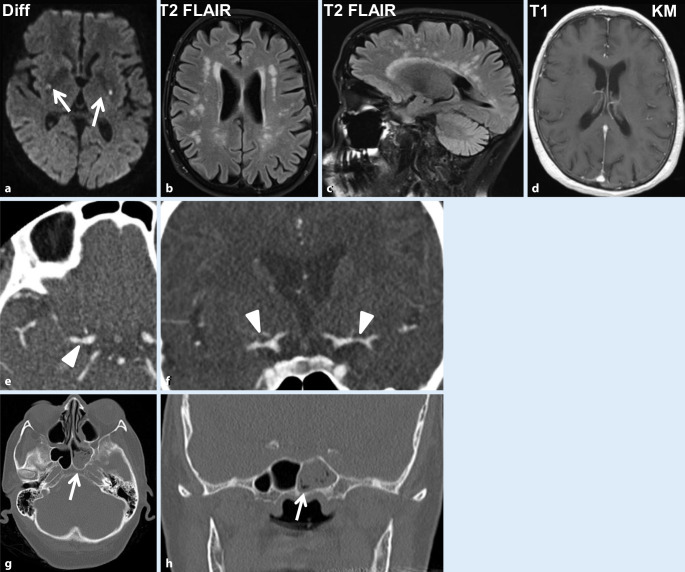

Klinisch-neurologisch stellte sich rasch nach stationärer Aufnahme ein Spontannystagmus nach rechts mit Drehschwindel ein, die Dysästhesien und Fußheberparesen zeigten sich unverändert. Im MRT des Schädels zeigten sich akute punktförmige lakunäre Ischämien in den Stammganglien beidseits sowie in der Medulla oblongata links in den diffusionsgewichteten Sequenzen. Ferner imponierten in den FLAIR-Aufnahmen bihemisphärische Marklagerläsionen, die kein Kontrastmittel aufnahmen (Abb. 1a–d). Die CT-Angiographie bestätigte bilaterale Gefäßkaliberschwankungen (Abb. 1e, f), die hochgradig verdächtig für eine intrazerebrale Vaskulitis waren. Die Neurographie konnte eine motorische axonale Neuropathie an beiden Nn. tibiales und peroneus objektivieren, die kalorische Testung eine Untererregbarkeit am rechten Ohr und die Tonaudiometrie eine Hochtonsenke rechts.

In der Liquoruntersuchung stellte sich eine entzündliche Konstellation mit erhöhter Leukozytenzahl (41 Leukozyten; Normwert <5 Leukozyten/µl) und erhöhtem Eiweiß (802 mg/l; Normwert <400 mg/l) dar. Es fanden sich keine oligoklonalen Banden im Liquor. Die serologische differenzialdiagnostische Abklärung ergab erhöhte Antikörpertiter für c‑ANCA (antineutrophile zytoplasmatische Antikörper 1:80; Normwert <1:20) und ebenso deutlich erhöhte Proteinase-3-Antikörper (634,1 CU; Normwert <20 CU). Hinweise für eine Nierenbeteiligung ergaben sich nicht.

Bei der Suche nach einer Affektion der Atemwege zeigte sich in der CT der Nasennebenhöhlen eine Sinusitis ethmoidalis, maxillaris und sphenoidalis (Abb. 1g, h). Im Thoraxröntgenbild wurden Verschattungen gesehen, die sich in der Thorax-CT als bipulmonale multiple knotige Verdickungen bis 3 cm Durchmesser bestätigten. Eine Biopsie des Nasennebenhöhlenseptums erbrachte den Nachweis chronischer und akuter gefäßassoziierter Entzündungsinfiltrate. Epitheloidzellige Granulome oder eosinophile Zellen konnten jedoch nicht objektiviert werden. Auf eine erneute Biopsie wurde aufgrund der doch eindeutigen Befundkonstellation verzichtet.

## Therapie und Verlauf

Nach interdisziplinärer Fallbesprechung und Feststellung der Diagnose einer Granulomatose mit Polyangiitis und peripherer sowie zentraler Beteiligung initiierten wir eine Thrombozytenaggregationshemmung mit ASS 100 mg und eine immunsuppressive Therapie. Nach einem 3‑tägigen intravenösen Methylprednisolonstoß (Dosis 250 mg/Tag) stellten wir auf 1 mg/kg Körpergewicht orales Prednisolon um. Zeitgleich begannen wir aufgrund der Schwere der Symptome eine Induktionstherapie mit Cyclophosphamid mit 500 mg i.v. pro Zyklus in einem Abstand von 2 Wochen. Es wurden insgesamt 6 Zyklen durchgeführt (Kumulativdosis 3000 mg Cyclophosphamid). Im Anschluss an die Cyclophophamidtherapie begannen wir eine körperoberflächenadaptierte remissionserhaltende Therapie mit dem Anti-CD20-Antikörper Rituximab (Dosis 375 mg/m^2^), die bis zum jetzigen Zeitpunkt etwas mehr als 2 Jahre nach Diagnosestellung weiterhin B‑Zell-gesteuert erfolgt. Die aktuelle Prednisolondosis beträgt derzeit 2,5 mg. Die Patientin hat sich unter der Therapie sehr gut stabilisiert und keine neuen neurologischen Symptome oder Läsionen im cMRT entwickelt. Die Dysästhesien der Fußsohlen und leichtgradigen Fußheberparesen (KG 4/5) bestehen zwar weiter fort, die Gangstörung hat sich jedoch deutlich gebessert.

## Diskussion

Unser Fall unterstreicht nochmals deutlich, dass alle Patienten, die sich mit subakuten neurologischen Symptomen, auch wenn diese auf das PNS beschränkt zu sein scheinen, ausführlich neurologisch untersucht werden sollten. Letztlich waren bei der Patientin mehrere Organsysteme durch die GPA betroffen. Dazu zählen PNS, ZNS, obere Atemwege, untere Atemwege sowie rechtes Ohr. Die weiteren Befunde aus entzündlichem Liquor, intrazerebralen Gefäßkaliberschwankungen und hoch spezifischen positiven Proteinase-3-Antikörpern [5, 6] unterstützten die Diagnose.

Konsentierte diagnostische Kriterien für die Diagnosestellung einer GPA existieren bis dato nicht [7]. 2012 wurde die Nomenklatur der systemischen Vaskulitiden revidiert [8, 9]. Nach diesen revidierten Chapel-Hill-Kriterien erfolgt weiterhin eine Einteilung nach Gefäßgröße. Während beispielsweise eine Riesenzellarteriitis oder Takayasu-Arteriitis den Großgefäßvaskulitiden zugerechnet werden, gehören die ANCA-assoziierten Vaskulitiden (AAV) zu den Kleingefäßvaskulitiden. Bei den AAVs unterscheidet man wiederum die Granulomatose mit Polyangiitis (GPA; Wegener) von der eosinophilen Granulomatose mit Polyangiitis (EGPA; Churg-Strauss) und der mikroskopischen Polyangiitis (MPA; [8, 9]). Positive Befunde für Proteinase-3-Antikörper, wie im geschilderten Fall, oder aber auch Myeloperoxidaseantikörper erleichtern die Zuordnung zu den AAVs [5, 6]. Proteinase-3-Antikörper sind dabei hinweisend für das Vorliegen einer GPA, während Myeloperoxidaseantikörper eher mit einer EGPA oder MPA assoziiert sind [7]. Während die GPA insbesondere obere und untere Atemwege befällt, finden sich bei der EGPA erhöhte Anteile an eosinophilen Leukozyten im Blut und in der Histologie. Dies war im vorliegenden Fall nicht gegeben.

Prinzipiell ist beim Verdacht auf eine Vaskulitis der Nachweis in einer Biopsie anzustreben. Hierfür eignen sich insbesondere der HNO-Trakt, aber auch Nieren, Haut oder Nerven bei entsprechendem Befall. Im vorliegenden Fall konnte durch eine Septumbiopsie eine Gefäßentzündung gesichert werde. Aber selbst wenn eine Biopsie keinen eindeutigen Befund bringt oder nicht durchgeführt werden kann, ist bei entsprechender Antikörperkonstellation und entsprechenden Symptomen eine AAV weiterhin möglich und sollte nicht zu einer Therapieverzögerung führen [7]. Trotz der bei unserer Patientin vorliegenden Schwerpunktneuropathie verzichteten wir auf eine zusätzliche Nervenbiopsie aufgrund der bereits erhobenen Befunde.

Neurologische Manifestationen (Tab. 1) bei einer GPA sind grundsätzlich selten bei Erstmanifestation, nehmen aber im Erkrankungsverlauf deutlich zu. In einer retrospektiven Analyse von GPA-Patienten fanden sich im Verlauf bei immerhin 11,7 % der GPA-Patienten ZNS-Symptome, wohingegen in einer prospektiven Analyse eine Beteiligung des peripheren Nervensystems bei bis zu 44 % der Patienten zu beobachten war [3, 10]. Neben Kopfschmerzen, akuten neurologischen Ausfällen jeder Art, Hirnnervenausfällen (auch bilateral) und subakut auftretenden peripheren Dysästhesien oder Paresen zählen auch Hörminderungen oder Sehstörungen oder ein zentraler Diabetes insipidus zu möglichen zentralen oder peripheren neurologischen Manifestationen. In der MRT sind sowohl der Nachweis akuter Schlaganfälle als auch ausgedehnte ältere Marklagergliosen wie im berichteten Fall möglich [3]. Zusätzlich können Zeichen einer Schwerpunktneuropathie (als vaskulitische Neuropathie) in der Neurographie nachweisbar sein [10, 11]. Die Hirnnervenausfälle können durch eine Granulomatose der basalen Hirnstrukturen verursacht werden [4], was bei unserer Patientin aber nicht der Fall war.

**Table 1:** 

Symptom	Diagnostik
Kopfschmerzen/Meningismus	cMRT, Angiographie, Liquorpunktion
Hirnnervenausfälle	cMRT, Liquorpunktion, SSEP, MEP, Elektronystagmographie
Schlaganfälle/Enzephalopathie/Epilepsie	cMRT, Angiographie, Liquorpunktion
Diabetes insipidus/Hypophyseninsuffizienz	cMRT, Hormonspiegel, endokrinologische Vorstellung
Hörminderung	HNO-Vorstellung, CT-NNH
Sehstörung	Augenärztliche Vorstellung, cMRT
Dysästhesien/Paresen	Neurographie mit Elektromyogaphie, ggf. Nervenbiopsie

Da die Prävalenz neurologischer Symptome im Erkrankungsverlauf der GPA deutlich zuzunehmen scheint, ist die Kenntnis dieser Entität für uns Neurologen essenziell. Eine immunsuppressive Therapie sollte je nach Schwere der Symptome und Ausprägung des Organbefalls eingeleitet werden. Für die Remissionsinduktion kommen entsprechend der rheumatologischen S1-Leitlinie für die AAVs zusätzlich zu Glukokortikoiden sowohl Cyclophosphamid als auch Rituximab infrage [7].
